# Habitat-adapted heterologous symbiont *Salinispora arenicola* promotes growth and alleviates salt stress in tomato crop plants

**DOI:** 10.3389/fpls.2022.920881

**Published:** 2022-08-08

**Authors:** Amayaly Becerril-Espinosa, Rosalba M. Hernández-Herrera, Ivan D. Meza-Canales, Rodrigo Perez-Ramirez, Fabián A. Rodríguez-Zaragoza, Lucila Méndez-Morán, Carla V. Sánchez-Hernández, Paola A. Palmeros-Suárez, Oskar A. Palacios, Francisco J. Choix, Eduardo Juárez-Carrillo, Martha A. Lara-González, Miguel Ángel Hurtado-Oliva, Héctor Ocampo-Alvarez

**Affiliations:** ^1^Departamento de Ecología, Centro Universitario de Ciencias Biológicas y Agropecuarias, Universidad de Guadalajara, Guadalajara, Mexico; ^2^Consejo Nacional de Ciencia y Tecnología, Mexico City, Mexico; ^3^Departamento de Botánica y Zoología, Centro Universitario de Ciencias Biológicas y Agropecuarias, Universidad de Guadalajara, Guadalajara, Mexico; ^4^Instituto Transdisciplinar de Investigación y Servicios, Centro Universitario de Ciencias Exactas e Ingenierías, Universidad de Guadalajara, Guadalajara, Mexico; ^5^Departamento de Producción Agrícola, Centro Universitario de Ciencias Biológicas y Agropecuarias, Universidad de Guadalajara, Guadalajara, Mexico; ^6^Facultad de Ciencias Químicas, Universidad Autónoma de Chihuahua, Chihuahua, Mexico; ^7^Facultad de Ciencias del Mar, Universidad Autónoma de Sinaloa, Mazatlán, Mexico

**Keywords:** *Salinispora* heterologous symbiont, marine actinobacteria, coral actinobacteria, plant salt stress, plant biostimulant, photoprotective response

## Abstract

To ensure food security given the current scenario of climate change and the accompanying ecological repercussions, it is essential to search for new technologies and tools for agricultural production. Microorganism-based biostimulants are recognized as sustainable alternatives to traditional agrochemicals to enhance and protect agricultural production. Marine actinobacteria are a well-known source of novel compounds for biotechnological uses. In addition, former studies have suggested that coral symbiont actinobacteria may support co-symbiotic photosynthetic growth and tolerance and increase the probability of corals surviving abiotic stress. We have previously shown that this activity may also hold in terrestrial plants, at least for the actinobacteria *Salinispora arenicola* during induced heterologous symbiosis with a wild Solanaceae plant *Nicotiana attenuata* under *in vitro* conditions. Here, we further explore the heterologous symbiotic association, germination, growth promotion, and stress relieving activity of *S. arenicola* in tomato plants under agricultural conditions and dig into the possible associated mechanisms. Tomato plants were grown under normal and saline conditions, and germination, bacteria-root system interactions, plant growth, photosynthetic performance, and the expression of salt stress response genes were analyzed. We found an endophytic interaction between *S. arenicola* and tomato plants, which promotes germination and shoot and root growth under saline or non-saline conditions. Accordingly, photosynthetic and respective photoprotective performance was enhanced in line with the induced increase in photosynthetic pigments. This was further supported by the overexpression of thermal energy dissipation, which fine-tunes energy use efficiency and may prevent the formation of reactive oxygen species in the chloroplast. Furthermore, gene expression analyses suggested that a selective transport channel gene, *SlHKT1,2*, induced by *S. arenicola* may assist in relieving salt stress in tomato plants. The fine regulation of photosynthetic and photoprotective responses, as well as the inhibition of the formation of ROS molecules, seems to be related to the induced down-regulation of other salt stress response genes, such as *SlDR1A*-related genes or *SlAOX1b.* Our results demonstrate that the marine microbial symbiont *S. arenicola* establishes heterologous symbiosis in crop plants, promotes growth, and confers saline stress tolerance. Thus, these results open opportunities to further explore the vast array of marine microbes to enhance crop tolerance and food production under the current climate change scenario.

## Introduction

Plant biostimulants (PBs) based on microorganisms have emerged as sustainable alternatives to traditional agrochemicals by enhancing plant stress tolerance and increasing crop yields and quality under suboptimal conditions. Nowadays, PBs are becoming essential components of strategies to mitigate the effects of climate change. In their native environments, plants live and grow with many symbiotic microorganisms. However, intensive cultivation practices and the indiscriminate use of fertilizers and agrochemicals, which at one point increased the ecological success of plants, have modified the natural microbiota of crop soils. Microorganism-based PBs take advantage of the symbiotic microorganisms that typically occur in nature and reintroduce them into empty crop soil, which enables plants to withstand environmental changes that threaten the global crop industry (Shameer and Prasad, 2018).

Currently, one of the principal problems that affects crop productivity is soil salinization. Soil salinization has increased due to the over-irrigation and over-fertilization of high-yield cropping systems, which have affected 931.67 million hectares worldwide as of 2018 (Mukhopadhyay et al., 2021). The resulting high salt concentrations in soils produce osmotic stress and cause imbalances in water and nutrient uptake by plants (Arif et al., 2020). Moreover, a high influx of Na^+^ ions into the plant cells results in the displacement of K^+^ ions from many enzymes and cofactors, which affects plant metabolism (Abdelaziz et al., 2019). Both ionic and osmotic stressors promote the accumulation of reactive oxygen species (ROS) and cause oxidative stress, which can damage DNA, proteins, and lipids (Shahid et al., 2020). These plant stressors affect germination, photosynthesis, growth, flowering, and many other physiological processes responsible for the low crop yields associated with saline soils (Parihar et al., 2015).

Plants have evolved complex survival responses to cope with salinity that involve coordination among many physiological and genetic processes, such as controlling water loss through stomata pore regulation, ion sequestration, metabolic and osmotic adjustments, and the activation of enzymatic and non-enzymatic mechanisms of the antioxidant system to maintain cellular redox homeostasis (Orsini et al., 2011; Abo-Ogiala et al., 2014; Adem et al., 2014; Geilfus et al., 2015; Soares et al., 2019). However, natural plant responses are not always sufficiently able to cope with stress. To our knowledge, no reliable process is available to fully restore saline soils (Shankar and Evelin, 2019). However, the inoculation of plants with certain microorganisms can increase their salt tolerance and enable them to be cultivated under saline stress conditions. Rodriguez et al. (2008) elegantly demonstrated a process, which they defined as habitat-adapted symbiosis (HAS), in which endophytic symbionts of native halophytes enable saline-sensitive plants to grow in saline soils. Since then, HAS strategies employing various halophytes from different saline habitats have been explored to relieve salt stress in salt-sensitive plant species. Some examples of HAS species that confer resistance to salt stress in plants are the halophilic bacteria *Stenotrophomonas rhizophila*, which inhabits the halophyte *Lygeum spartum* that is native to the desert environments of Algeria; *Bacillus atrophaeus*, which is an endophyte of the halophytes *Suaeda mollis* and S*alsola tetrandra* that are native to Algeria Salt Lakes; *Jejubacter calystegia*e, which is found in the halophyte *Calystegia soldanella* that is found on sandy beaches in South Korea; and many fungus-like microorganisms like *Neocamarosporium chichastianum*, *N. goegapense*, and *Periconia macropinosa*, which has been isolated from the desert halophyte *Seidlitzia osmarinus* collected at Hoz-e Soltan Salt Lake in Iran (Jiang et al., 2021; Kerbab et al., 2021; Moghaddam et al., 2021; Dif et al., 2022). Some of these HAS species have been successfully employed in PB formulations.

Recently, it was shown that the marine actinobacteria *Salinispora arenicola* could establish a symbiotic relationship with seedlings of the wild-tobacco *Nicotiana attenuata* under *in vitro* conditions (Ocampo-Alvarez et al., 2020) and relieve saline stress during germination and early growth. As *S. arenicola* is a saline habitat symbiont that relieves saline stress in the saline-sensitive *N. attenuata*, it partially fulfills the HAS definition. However, the native habitat of *S. arenicola* is marine, and it is found within *Porites* corals where it establishes what seems to be three symbiotic trophic interactions between the coral and *Symbiodinium* sp., a phototrophic alga. Therefore, *S. arenicola* is better defined as a habitat-adapted heterologous symbiont (HAHS). Nevertheless, the potential of this HAHS species to relieve salt stress and any associated mechanisms remain unexplored in terrestrial plants of agricultural interest.

All HAS possess specific mechanisms to ameliorate saline stress in plants. A common HAS mechanism is the production of osmolytes, such as proline, trehalose, glycine betaine, or exopolysaccharides, which directly ameliorate osmotic stress by decreasing the internal osmotic potential of plants cells (Bano and Fatima, 2009; Upadhyay et al., 2012; Timmusk et al., 2014; Upadhyay and Singh, 2015). Other HAS mechanisms involve activating antioxidant systems, such as enzymes, biochemical compounds, and secondary metabolites that counteract the induced oxidative stress (Hashem et al., 2016). Moreover, several HAS also regulate phytohormone activity or biosynthesis. For example, ACC (1-aminocyclopropane-1-carboxylate) deaminase activity lowers the level of the stress-related phytohormone ethylene, thus avoiding the down-regulation of genes involved in plant growth (Glick et al., 2007). Another mechanism to cope with salinity in plants is the overexpression of specific ionic channels that counterbalance the K^+^/Na^+^ proportion, such as high-affinity Na^+^ and K^+^ transporters (i.e., High-affinity K^+^, *HKT*; Na^+^/K^+^ antiporters, *NHX*; or inward-rectifier K^+^ channel/transporters, *AKTs*; Arif et al., 2020). Different mechanisms produce different levels of protection, which may lead to improved germination, photosynthesis, growth, flowering, or yield under saline conditions.

Tomato (*Solanum lycopersicum*) is an agriculturally important crop, and its responses to salt stress have been abundantly reported. Some of the most recognizable effects of salinity in tomato plants are reduced growth; decreased dry weights (DW) of roots, stems, and leaves; and leaf senescence (Munns, 2002; Zribi et al., 2009). Growth reduction is a consequence of Na^+^ accumulation due to a decline in the water potential, which affects photosynthetic activity (Iyengar and Reddy, 1996; Hanachi et al., 2014). Stomatal closure is probably the first plant protection response against desiccation and a direct factor that immediately controls carbon fixation in the case of C3 plants. Long-term exposure to saline treatments in tomatoes has been shown to result in a notable and progressive decline in photochemical processes and a notable increase in non-photochemical quenching (NPQ) that protects the plant from an overreduction of the quinone electron acceptor (QA; Zribi et al., 2009).

In this study, the properties of the marine actinobacteria *S. arenicola* as a PB and bio-protector under normal and saline conditions were evaluated in tomato plants. First, the nature of the interaction between the tomato plants and *S. arenicola* was established. Then, the potential of this actinobacteria to relieve induced salt stress during germination and plant growth was explored based on the assumption that the HAHS *S. arenicola* may possess specific activities that could alleviate salt stress in tomato plants. Finally, the photosynthetic performance, pigment composition, osmolyte content, and changes in the antioxidant properties of tomato plants were explored while scouting for changes in the expression of genes potentially involved in different mechanisms induced by salt stress conditions. Overall, our results support the potential of this marine actinobacterium to be used as a crop PB and protective agent against salt stress.

## Materials and methods

### Biological samples

*Salinispora arenicola* strain X29 (GenBank accession number MT002753^1^) was isolated from *Porites lobata* obtained from the Tropical Central Pacific coral reef system (19° 5′ 55.21″ N, 104° 23′ 24.47″ W and 19° 3′ 28.87″ N, 104° 15′ 40.25″ W) and cryopreserved at −70°C in 15% glycerol (Ocampo-Alvarez et al., 2020) until use.

The bacterial suspension used to treat Tomato plants was prepared as follows: *S. arenicola* X29 was cultivated on A1 medium (Mincer et al., 2002) under agitation at 210 rpm and 28°C for 5 days and then centrifuged at 10,000 × *g* for 5 min. The supernatant was discarded, and the pellet was weighed and resuspended with distilled water to a final concentration of 1 mg/mL.

**FIGURE 1:**
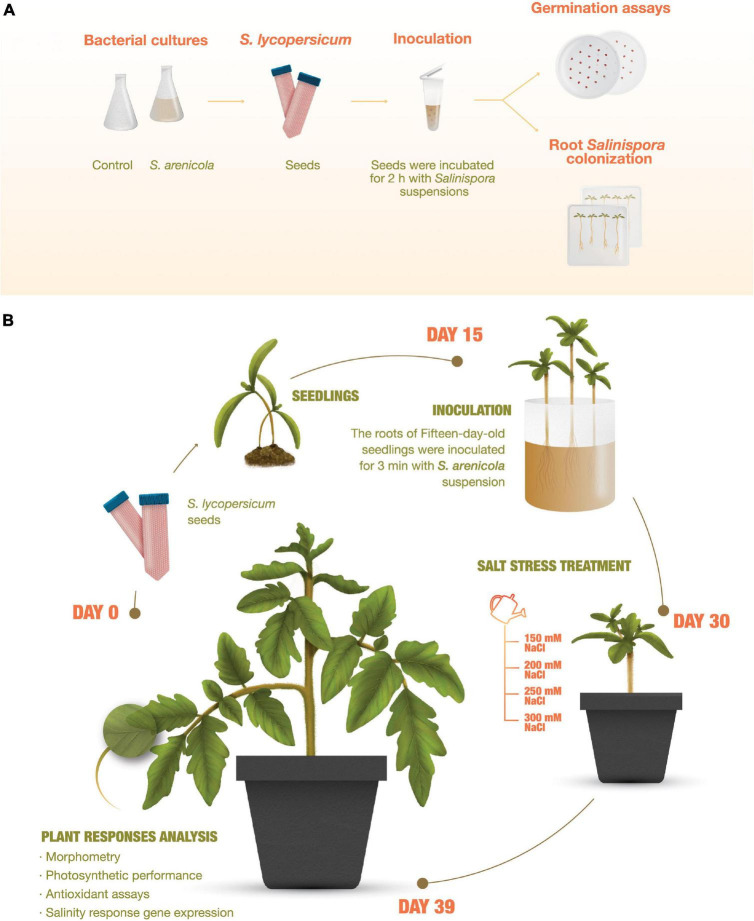
Experiment outline. **(A)** Root *Salinispora* colonization and germination assays of tomato (*Solanum lycopersicum*) plants. Seeds were sterilized, directly inoculated with *S. arenicola* X29, and sown and cultured in phytagel-agar plates. Growth and plant response analyses. **(B)** Tomato seedling roots were inoculated on day 15. On day 30, the plants were treated for 8 days with increasing NaCl concentrations and analyzed on day 39.

All experiments were conducted using tomato plants (*S. lycopersicum* L. cv. Rio Fuego; Kristen Seed, San Diego, CA, United States). Tomato seeds were cleaned by immersion on a 10% v/v household soapy solution for 1 min and washed thrice with sterile distilled water. They were then disinfected with 3% sodium hypochlorite for 10 min and washed thrice with sterile distilled water. Subsequently, the seeds were either treated with *S. arenicola* X29 or not and then sown in phytagel-agar media for the germination and fluorescence *in situ* hybridization (FISH) experiments or with peat moss in germination trays and later treated at the seedling stage with *S. arenicola* X29 for subsequent plant response analyses (Figure 1). The plants were grown at all times in an artificial climate growth chamber (Thermo Fisher Scientific, Waltham, MA, United States) at 28/17°C and with a 16:8 h day/night photoperiod (155–300 μm s^–1^ m^–2^).

### Root *Salinispora* colonization

Tomato seeds were cleaned as previously described and incubated for 2 h in 3 mL of *S. arenicola* X29 culture or water (i.e., control) right after cleaning (Figure 1). Then, the seeds were sown in sterile plates with solid plant growth media [6 g L^–1^ phytagel (Sigma-Aldrich, St. Louis, MO, United States) and 3.2 g L^–1^ Gamborg’s B-5 Basal Medium with Minimal Organics (Sigma-Aldrich)], with or without 100 mM NaCl (SIGMA, Merck KGaA, Darmstadt, Germany). Seeds were then incubated at 28/17°C with a 16:8 light/dark photoperiod (155–300 μm s^–1^ m^–2^) in a growth chamber. After 30 days, the roots were harvested, fixed with formaldehyde, and stored at −20°C until needed for Giemsa staining and FISH hybridization.

Giemsa-stained roots were then observed under a light microscope (Olympus BX41; Olympus, Tokyo, Japan; Muresu et al., 2010). FISH was performed according to the methods of de-Bashan et al. (2011) and Palacios et al. (2019). Three probes labeled with FAM dye, which have been previously reported, were used to target eubacteria: EUB-338-I (5′GCT GCC TCC CGT AGG AGT-3′), EUB-338-II (5′-GCA GCC ACC CGT AGG TGT-3′), and EUB-338-III (5′ GCT GCC ACC CGT AGG TGT-3′; Amann et al., 1990; Daims et al., 1999). The samples were visualized with a BX41 epifluorescence microscope (Olympus) using a FITC filter at 550/570 nm (excitation/emission; Olympus America, Melville, NY, United States).

### Germination assays

The tomato seed germination assay was conducted according to the standard protocol of the international society for seed bioassays (ISTA), which was modified by adding the actinobacteria or A1 medium (i.e., controls) as previously described. Approximately 800 surface-sterilized seeds were mixed with 3 mL of *S. arenicola* X29 suspension culture or A1 medium (control) for 2 h (Figure 1). Under sterile conditions, the seeds were transferred into Petri dishes containing wet Whatman no. 5 filter paper (Whatman International Limited, Maidstone, United Kingdom).

The salt stress experiment was run with 8 mL of 100 mM NaCl or sterile distilled water (control). Four groups, each containing 100 seeds, were included in each experiment. The seeds were incubated in a growth chamber at 28°C with a 16:8 light/dark photoperiod (155–300 μm/s/m^2^). Seed germination was measured every day for 8 days and only when the radicle was at least 2 mm long. The germination percentage was determined using the following formula:


$$\%Germination=\frac{n\,u\,m\,b\,e\,r\,o\,f\,g\,e\,r\,m\,i\,n\,a\,t\,e\,d\,s\,e\,e\,d\,s}{t\,o\,t\,a\,l\,n\,u\,m\,b\,e\,r\,o\,f\,s\,e\,e\,d\,s}\times 100$$

Germination was followed daily for 8 days after sowing. The seed germination data were fitted to the Gompertz equation: $f=a\,\left(e\,x\,p^{-e\,x\,p\,\left[-\left(\frac{d-d_{0}}{b}\right)\right]}\right)$, where *a* represents the maximum germination percentage and *d_0* represents the half time of maximum germination. Germinated seeds were grown for five additional days (13 days after sowing), and the morphological properties of the seedlings were analyzed. Specifically, the plumule and radicle lengths (cm) of the seedlings were measured, and the dry weight of the seedlings was determined after oven drying at 50°C to a constant weight. Nitrogen, carbon, and polyphenol content were determined in seedlings to evaluate any nutritional or antioxidant benefits induced by the actinobacteria.

Nitrogen and organic carbon percentages were determined according to the methodology established by the Association of Analytical Chemists (955.04; Association of Official Analytical Chemist, 1990). Total polyphenol content was evaluated with the Folin-Ciocalteu colorimetric method with a gallic acid standard (Singleton and Rossi, 1965).

### Treatment of plants with *Salinispora arenicola* for growth, photosynthesis, metabolite, antioxidant capacity, and gene expression analysis

Tomato seeds were germinated in peat moss as previously described without further treatment. Fifteen-day-old seedlings were washed with distilled water to remove peat moss. The roots were incubated for 3 min in the *S. arenicola* X29 suspension at a bacteria concentration of 1 mg/mL or water (control). The treated seedlings were transplanted into pots with 500 g of soil mixture composed of vermiculite, peat moss, and soil (1v:1v:1v; Figure 1).

### Salt stress treatment

Fifteen days after treatment with the *S. arenicola* suspension, a set of tomato plants was irrigated with increasing concentrations of saline water (150, 200, 250, and 300 mM NaCl) every second day. Photosynthetic performance and morphological parameters were analyzed for all treatments 9 days after inducing salt stress with saline water irrigation (Day 39, Figure 1). Whole leaf samples were collected, flash-frozen with liquid nitrogen, and stored at −70°C for metabolite, pigment, enzyme, or gene expression analyses.

### Photosynthetic performance analysis

Chlorophyll-*a* fluorescence was measured with a non-intrusive pulse-amplitude modulated (PAM) chlorophyll-fluorometer (Junior-PAM, Heinz Walz GmbH, Effeltrich, Germany). All photosynthetic parameters from the fluorescence measurements were obtained as described by Schreiber (2004) (Supplementary Table 1). Two standard protocols (i.e., induction curves and rapid light response curves) were used to obtain all photosynthetic parameters for our experiments. Both protocols were performed on the second youngest, attached, fully expanded, and healthy leaves of at least six plants per treatment. The light guide of the Junior-PAM was positioned at the midpoint of the adaxial side of the leaf for both fluorescence protocols. The protocols were initiated after 30 min of dark adaption.

The NPQ induction curves began with a saturating pulse of 10,000 μmol photons m^–2^ s^–1^ (800 ms) to measure maximum fluorescence when all PSII reaction centers were closed. This pulse was followed by 40 s of darkness to reestablish a basal F_0_ measurement, after which the leaf was subjected to 20 min of high actinic light (1,500 μmol photon m^–2^ s^–1^). Saturating light pulses (SP) were emitted during this lighting period every 30 s. The rapid light curves (RLCs) were set with 12 light levels (each turned on for 30 s): 25, 45, 65, 90, 125, 190, 285, 420, 625, 820, 1,150, and 1,500 μmol photon m^–2^ s^–1^. As in the induction curve experiments, the RLCs began with a saturating light pulse of 10,000 μmol photons m^–2^ s^–1^ (800 ms) to measure maximum fluorescence when all PSII reaction centers were closed. All experiments began early in the morning and were completed before noon. The induction curve and RLC parameters were obtained from the automatic data output of the Junior PAM software.

### Photosynthetic pigments

Photosynthetic pigments were extracted and measured using the method described by Zapata et al. (2000) following the recommendations of Osuna-Ruíz et al. (2019). Pigments were extracted from 300 mg of fresh frozen leaves in 90% acetone for 24 h at 4°C and then centrifuged at 3,220 × *g* for 10 min. The supernatants were recovered and stored at −20°C until use. Pigment quantification was performed with a 1200 Infinity HPLC system (Agilent Technologies, Santa Clara, CA, United States) coupled to a diode array detector (DAD) with a C-8 column (ZORBAX 4.6 mm × 100 mm, 3.5 μm particles). The mobile phases consisted of eluent A (methanol:acetonitrile:pyridine 0.25 M, 50:25:25, v:v:v) and eluent B (acetonitrile:acetone, 80:20, v:v) delivered with the following gradient scheme (A%/B%, min): 100/0, 0; 60/40, 22; 5/95, 28; 5/95, 38; and 0/100, 40. The flow rate was fixed at 1 mL per min. All solvents were HPLC grade (Tedia, OH, United States). Pigments were identified by comparing their retention times with standards (DHI, Hørsholm, Denmark). Pigment coefficients were calculated to evaluate the photosynthetic and photoprotective status of whole-treated plants. Total chlorophyll was obtained by the sum of Chl *a* + Chl *b* and was used as a proxy for photosynthetic capacity. The chlorophyll ratio was obtained by dividing Chl *a* by Chl *b* and was used as an estimator of LHCII antenna size and the light acclimation state. The pool size of the xanthophyll cycle pigments (∑*XC*) was obtained by the sum content of the three xanthophyll pigments [i.e., violaxanthin (Vx), antheraxanthin (Ax), and zeaxanthin (Zx)] and was used to estimate the photoprotective capacity to dissipate thermal energy. The percentage of xanthophyll cycle pigments in deepoxidated state DEPS was obtained by the formula $\left(\frac{\left[Z_{x}\right]+0.5\,\left[A_{x}\right]}{\sum X\,C}\right)\times 100$ and was used to assess the actual photoprotective state of the ∑*XC* pool. The β-carotene:total chlorophyll ratio,$\frac{\beta -c\,a\,r}{C\,h\,l\,a+b}$, was used as a proxy of the antioxidant capacity.

### Total phenol, flavonoid, proline, and reducing sugar content

Phenol, flavonoid, and reducing sugar content were determined from acidified ethanol extractions conducted with fresh frozen leaves (six plants per treatment) that were pulverized and lyophilized (ILSHIN BIOBASE Table Top Freeze Dryer). A total of 25 mg of lyophilized leaves was extracted in 70% ethanol (acidified with 0.1% HCl) for 24 h at 4°C in the dark. The mixture was centrifuged at 4°C for 20 min at 10,000 × *g*, and the supernatant was collected for metabolite analysis.

Total polyphenol content was determined using the Folin-Ciocalteu (F-C) colorimetric method adjusted for micro-determination (Cao et al., 2020) with some modifications. A 100-μL aliquot of the sample extract and 500 μL of F-C reagent were mixed and incubated at room temperature for 5 min. Afterward, 400 μL of Na_2_CO_3_ (15%) was added to the solution. The mixture was incubated at 45°C for 10 min. A total of 200 μL of the reaction mixture was placed into a 96-well plate. The absorbance was measured at 730 nm in a microplate reader (Multiskan™ GO Microplate Spectrophotometer, Thermo Fisher Scientific, Waltham, MA, United States) after mixing and an 80-min incubation in the dark. A standard curve using gallic acid was recorded (10–200 μg/mL). Phenol content was expressed as gallic acid equivalents (GAE, mg) per gram of DW (mg GAE/g DW).

The reducing sugar assay was conducted according to the methodology of Negrulescu et al. (2012) with some modifications. A 100-μL aliquot of the sample extract was added to 1,000 μL of DNS reagent (3,5-dinitro salicylic acid, Sigma Aldrich) and mixed. The samples were incubated for 10 min at 100°C. Subsequently, the mixture was allowed to cool to room temperature for 4 min, and 200 μL was placed in a microplate. The absorbance was read at 540 nm with the Multiskan™ GO microplate reader (Thermo Fisher Scientific). A glucose calibration curve ranging from 1–10 mg/mL was used.

The proline assay was conducted as described by Palmeros-Suárez et al. (2015). Briefly, leaves were harvested, flash-frozen in liquid nitrogen, pulverized, and lyophilized. A total of 25 mg of powdered tissue from each sample was mixed with 100 μL of the proline extraction buffer [80% ethanol, 100 mM HEPES *N*-(2-Hydroxyethyl)piperazine-N′-(2-ethane sulfonic acid) buffer at pH 7.4, and 5 mM MgCl_2_]. The mixture was incubated at 4°C for 10 min with shaking and then centrifuged at 10,000 × *g* at 4°C for 10 min. The supernatant was transferred to a new tube and concentrated by vacuum centrifugation using a Maxi Dry Lyo (Heto-Holten, Allerød, Denmark). The precipitate was dissolved in 100 μL of HEPES buffer (100 mM, pH 7.4) with MgCl_2_ (5 mM). A 100-μL aliquot of the former solution was added to 200 μL of a solution containing glacial acetic acid 60% (v/v), ethanol 20% (v/v), and ninhydrin 1% (w/v). The mixture was boiled at 95°C for 20 min, cooled to room temperature, and the optical density was recorded at 520 nm in the Multiskan™ GO microplate reader (Thermo Fisher Scientific).

### Antioxidant capacity

Antioxidant capacity was measured according to the methodology of Brand-Williams et al. (1995) following the recommendations of Rubio-Ochoa et al. (2019). A total of 100 μL of each sample extract and 900 μL of 0.1 M of the stable radical 2,2-diphenyl-1-picrylhydrazyl (DPPH^∙^ in 70% ethanol) were mixed for 30 min at room temperature in the dark. Subsequently, a 250-μL aliquot of each sample was placed in the 96-well Multiskan™ GO microplate (Thermo Fisher Scientific) and read at 515 nm. The result was expressed in micromol equivalents of Trolox per gram of tissue (DW) using a calibration curve ranging from 1–1,000 μM/mL.

### Salinity responsive gene expression assay

Total RNA was extracted from leaves obtained from a pool of three plants using TRIzol Reagent (Invitrogen, Carlsbad, CA, United States) following manufacturer recommendations. The cDNA was obtained from 2 μg of RNA using GoScript reverse transcriptase (Promega, Madison, NJ, United States) and oligo-dT (15) primers. qPCR amplifications were performed using SYBR^®^ Green detection in 96-well plates in a StepOnePlus System (Applied Biosystems, Foster City, CA, United States). Reactions were prepared in a total volume of 15 μL with 1.5 μL of cDNA template (1:10), 7.5 μL SYBR^®^ Select Master Mix (Applied Biosystems), and forward and reverse gene-specific primers (300 nM). The cycling conditions were set as follows: initial denaturation step at 95°C for 5 min, followed by 40 cycles of denaturation at 95°C for 10 s, and annealing at 60°C for 30 s. A melting curve analysis was used to evaluate reaction specificity. The RT-qPCR system software automatically determined the baseline and cycle threshold (Ct). Relative expression was calculated using a comparative cycle threshold method (Livak and Schmittgen, 2001) and *SlGADPH* and *SlEF1α* as endogenous reference genes. Primers for *SlGADPH* and salinity responsive genes were reported by Devkar et al. (2020) and *SlEF1α* by Wang et al. (2020) (Supplementary Table 2).

### Statistical analysis

The data sets were analyzed by a one-way ANOVA and *post hoc* Tukey HSD tests after both normality and homogeneity of variance were tested with Shapiro–Wilk and Bartlett tests, respectively. When these assumptions were not met, a permutational ANOVA (PERMANOVA) was used. All statistical analyses were conducted in Sigma Plot v. 12 (Systat Software, Inc., Chicago, IL, United States) and PRIMER + PERMANOVA software (Version 7.0.13; PRIMER-e, Plymouth, United Kingdom).

## Results

### Tomato root colonization by *Salinispora arenicola*

**FIGURE 2:**
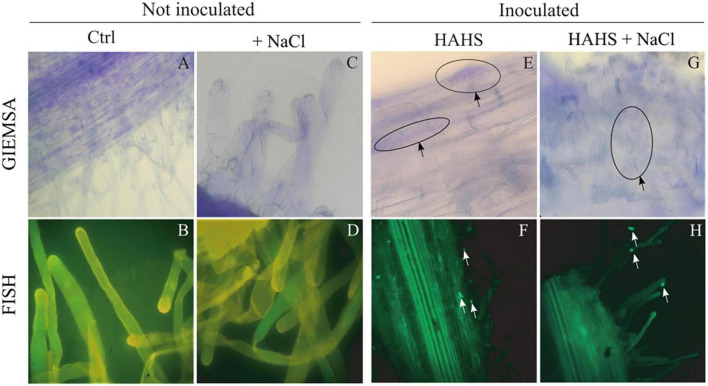
Colonization of *Salinispora arenicola* on tomato root hairs. Samples underwent Giemsa staining **(A,C,E,G)** and Fluorescence *in situ* Hybridization (FISH; **B**,**D**,**F**,**H**). Arrows indicate the presence of habitat-adapted heterologous symbiont (HAHS) bacterial cells in samples of plants inoculated under control (Ctrl; without NaCl) and NaCl conditions (100 mM).

In a previous study (Ocampo-Alvarez et al., 2020), a close interaction between *S. arenicola* and *Nicotiana attenuata* roots was observed *in vitro*. To confirm the presence of such interactions in tomato plants in this study, tomato seeds inoculated with *S. arenicola* and controls (bacteria-free) were grown under either saline or salt-free conditions and analyzed by Giemsa staining and FISH (Figure 2). Colonization of *S. arenicola* was observed in the central zone of the seedling roots. Under salt-free conditions, we found the bacteria interacting with the epidermal cells of roots (Figures 2E,F). Interestingly, bacterial colonization was more markedly observed under saline conditions on root hairs (Figures 2G,H). The control seedlings showed no presence of any bacteria (Figures 2A–D), which confirms that *S. arenicola* interaction occurs in tomato plants and that the site of this interaction changes from epidermal cells to root hairs based on salinity conditions. As *S. arenicola* is a bacteria found in saline habitats with the capacity to sustain a heterologous symbiotic relationship with plant roots, hereinafter it is referred to as a HAHS.

### *Salinispora arenicola* increases the germination percentage of tomato seeds under salt stress conditions

**FIGURE 3:**
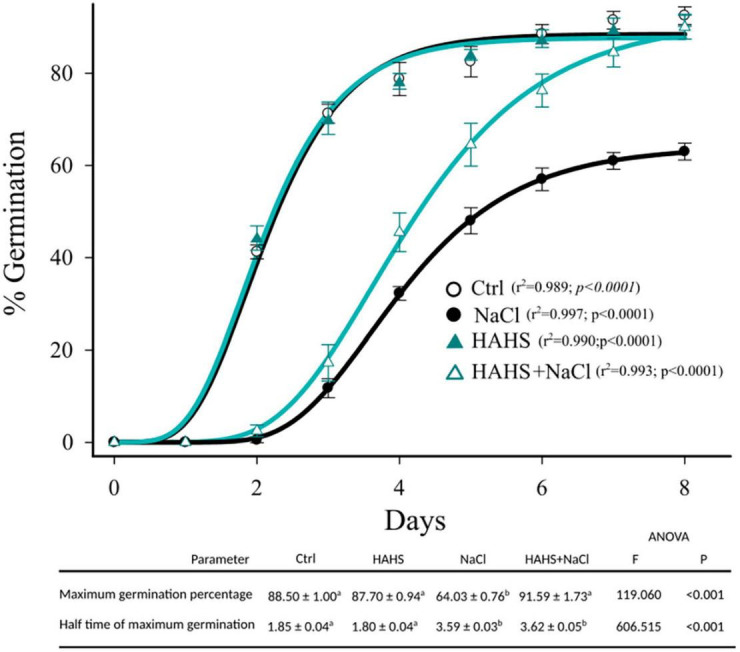
Germination kinetics of HAHS-treated and control tomato seeds under saline conditions. Symbols represent the daily average germination percentage of four replicates with 100 seeds. Seed germination data were fitted to the Gompertz equation: $f=a\,e^{\left(-e^{-\left(\frac{d-d\,0}{b}\right)}\right)}$. Seeds inoculated under control (Ctrl) or habitat-adapted heterologous symbiont (HAHS) conditions with water free of NaCl or with 100 mmol NaCl (+NaCl) irrigation. Statistical significance was analyzed by a univariate analysis of variance. Lowercase letters denote statistical differences (pairwise test) among treatments for each parameter.

Once the interaction between *S. arenicola* and the tomato plants was confirmed, the effects of this HAHS on germination competence were evaluated (Figure 3). With salt-free irrigation, no differences were observed in germination kinetics between the HAHS-treated and control seeds with ∼90% seed germination. Salinity delayed the germination kinetics in both HAHS-treated (HAHS+NaCl) or HAHS-free seeds (NaCl). However, although NaCl treatment caused a delay in seed germination, HAHS-treated seeds reached the maximum germination percentage (∼90%) at the end of the germination assay as did the control seeds. In comparison, HAHS-free seeds with NaCl treatment only reached ∼60% germination. Salt stress affected tomato seed germination, but treatment with *S. arenicola* alleviated this stress by the end of the experiment.

Additionally, in the germinated seedlings, we measured %N, %C, and polyphenol (mg/Kg) content and the radicle and plumule heights on day 13 (Table 1). Salinity decreased the plumule and radicle lengths of both HAHS-treated and HAHS-free seedlings. Interestingly, treatment with *S. arenicola* significantly increased both length measurements under conditions of no salt stress. Seedling plumule and radicle lengths correlated nicely with nitrogen content (Pearson r ≈ 0.8), although the relationships with polyphenols and carbon showed different patterns. Polyphenol content decreased in the control tomato seedlings under salt stress conditions but increased in the plants treated with *S. arenicola* under salt stress. The high polyphenol content in HAHS-treated tomato seedlings could be related to the enhanced antioxidant activity that protected tomato seeds during germination. This is supported by the high correlation between the germination percentage and polyphenol content (Pearson correlation: *r* = 0.95). Furthermore, high correlations were found between %N and %C content and germination (Pearson correlation: *r* = 0.97, 0.94, respectively).

**TABLE 1:** Growth and biochemical characteristics of habitat-adapted heterologous symbiont (HAHS)-treated (B) and control (C) tomato seedlings under salt stress conditions.

	Ctrl	HAHS	NaCl	HAHS + NaCl
Plumule (cm)	6.61 ± 0.24^b^	9.29 ± 1.18^c^	3.84 ± 0.46^a^	4.51 ± 0.40^a^
Radicle (cm)	6.00 ± 0.40^b^	7.35 ± 0.24^c^	3.40 ± 0.21^a^	3.96 ± 0.64^a^
% N	5.09 ± 0.01^b^	5.19 ± 0.01^b^	3.74 ± 0.03^a^	4.72 ± 0.09^c^
% C	43.42 ± 0.09^b^	38.72 ± 0.14^d^	31.20 ± 0.30^a^	41.02 ± 0.19^c^
Polyphenols (mg/kg)	901.81 ± 6.37^b^	600.01 ± 1.71^d^	356.97 ± 5.52^a^	1,582.53 ± 14.17^c^

Ctrl, control; HAHS-free; HAHS = S. arenicola; NaCl = HAHS-free and NaCl condition (100 mM); and HAHS + NaCl = S. arenicola and NaCl condition (100 mM).

### Growth promoting activity of *Salinispora arenicola* with saline and salt-free irrigation

**FIGURE 4:**
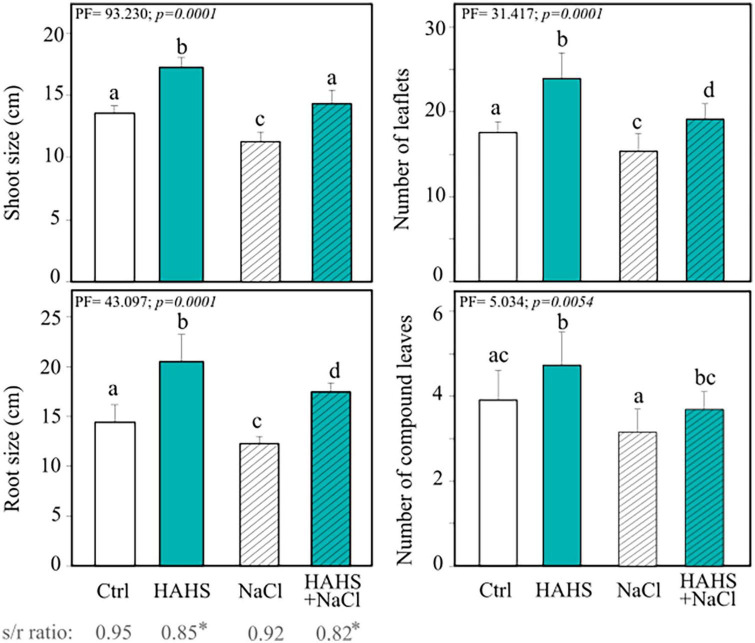
*Salinispora arenicola* enhances the growth of tomato plants under normal and salt stress conditions. Phenotype measurements of 39 DAG plants grown with either NaCl-free irrigation or saline irrigation that were either inoculated with a habitat-adapted heterologous symbiont (HAHS) bacterium or not (control; Ctrl). Statistical significance was determined using a permutational ANOVA (PERMANOVA; *p = 0.001*). Lowercase letters denote statistical differences (pairwise test) among conditions.

Tomato plant seedlings (15 days after germination; DAG) were submerged in either an *S. arenicola* suspension (HAHS-treatment) or water (control) before being transplanted into pots with soil and allowed to grow for 15 more days (Figure 1B). Afterward, groups of plants of each treatment were irrigated for 8 days with increasing NaCl concentrations up to 300 mM, which resulted in another two treatments: HAHS+NaCl and NaCl. Plant response parameters (i.e., plant length from the bottom to the main apex and the number of leaflets) were recorded 1 day after the last irrigation treatment (day 9 after the first irrigation). Results showed a clear and significant effect of *S. arenicola* in promoting tomato plant growth (Figure 4). With simple irrigation (salt-free), we found increases in growth of 27.2% in the shoots and 42.3% in the roots of plants treated with *S. arenicola* compared to those of the controls. In addition, leaflets and compound leaves increased in number by 20.9% and 36.3%, respectively (Figure 4).

Saline irrigation showed a slight decrease in all analyzed phenotypes for both control and HAHS-treated plants (Figure 4). Interestingly, HAHS-treated plants under salt stress conditions grew higher than control plants grown without saline stress, with a slight difference in shoot length (5.7%) and a notable difference in root length (21.1%; *P* = 0.001). Moreover, we still observed that HAHS-NaCl plants substantially outgrew bacteria free-plants under saline conditions (NaCl), with 26.8% and 42.2% differences in shoot and root lengths, respectively, and differences of 17.1% and 24.6% in the number of leaflets and compound leaves, respectively (Figure 4). Overall, although growth was affected by salinity in both control and HAHS-treated plants, *S. arenicola*-treated plants outperformed the control plants, which further suggests induced salt-tolerance activity by the actinobacteria.

### *Salinispora arenicola* enhances photosynthetic performance and photoprotection

**FIGURE 5:**
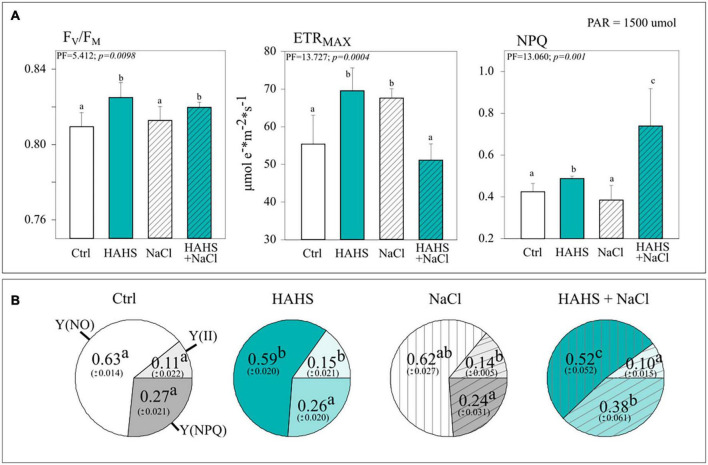
*Salinispora arenicola* enhances quantum yield and photoprotection under saline stress. **(A)** Photosynthethic performance parameters and **(B)** Yield coefficients of the absorbed energy distribution. Parameters were measured with a non-intrusive pulse-amplitude modulated (PAM) device (*n* = 6) 39 days after germination (DAG) in plants grown with either NaCl-free or saline irrigation and inoculated with a habitat-adapted heterologous symbiont (HAHS) bacteria (turquoise bars) or not (control; C, white bars). F_V_/F_M_, Maximum photochemical quantum yield of PSII; ETR_MAX_, Maximum electron transfer rate; NPQ, Stern-Volmer type non-photochemical fluorescence quenching; Y(NO), Quantum yield of non-regulated heat dissipation and fluorescence emission; Y(II), Effective photochemical quantum yield of PSII; Y(NPQ), Quantum yield of light-induced non-photochemical fluorescence quenching. Statistical significance was determined separately for each irrigation scheme using a univariate analysis of variance (ANOVA; *p =* 0.001). Lowercase letters denote statistical differences (pairwise test) among conditions.

In line with the growth results obtained with simple water irrigation, HAHS-treated plants exhibited enhanced photosynthetic performance compared to HAHS-free plants in the form of an ∼25% larger electron transport rate (ETR_MAX_), a marginally but statistically significant increase in the maximum photochemical quantum yield of PSII (F_V_/F_M_; c. 2%), and ∼15% higher dissipation of absorbed energy in the PSII antenna through a thermal dissipation pathway (NPQ; Figure 5A).

The yield coefficients of the absorbed energy distribution [effective photochemical quantum yield, Y(II); the non-regulated quantum yield of fluorescence emission from PSII, Y(NO); and light-regulated quantum yield of non-photochemical fluorescence quenching, Y(NPQ)] confirmed the bacterial benefits to photosynthetic performance. HAHS treatment induced a 40% increase of Y(II) compared to that of the control plants and a drop in the non-regulated Y(NO) and regulated Y(NPQ) energy distribution pathways in HAHS-treated compared to those of HAHS-free plants. This points to an increased capacity of HAHS-treated plants to allocate light-derived energy into the carbon assimilation pathway induced by the actinobacteria (Figure 5B).

Interestingly, when plants were irrigated with a saline solution, the photosynthetic parameters followed an interesting pattern of change with respect to those observed under non-stressed conditions in HAHS-free and HAHS-treated plants (Figure 5A). For HAHS-free plants, salt-stress induced no effect on F_V_/F_M_, although a reduction of ∼9% in NPQ and an increase of ∼20% in ETR_MAX_ were observed. On the other hand, HAHS-treated plants showed no increase in F_V_/F_M_, but in contrast, a significant increase in NPQ (∼74%) and a decrease in ETR_ MAX_ (∼7%) under saline stress was observed when compared to those of the control plants. This agrees with the observed changes induced by saline stress in yield coefficients, which showed a decrease in the proportion of Y(NO) of ∼17%, a marginal reduction in Y(II) of ∼7%, and a high increase in Y(NPQ) of ∼42% in HAHS-treated plants (Figure 5B).

### Photosynthetically related pigments and metabolites

**FIGURE 6:**
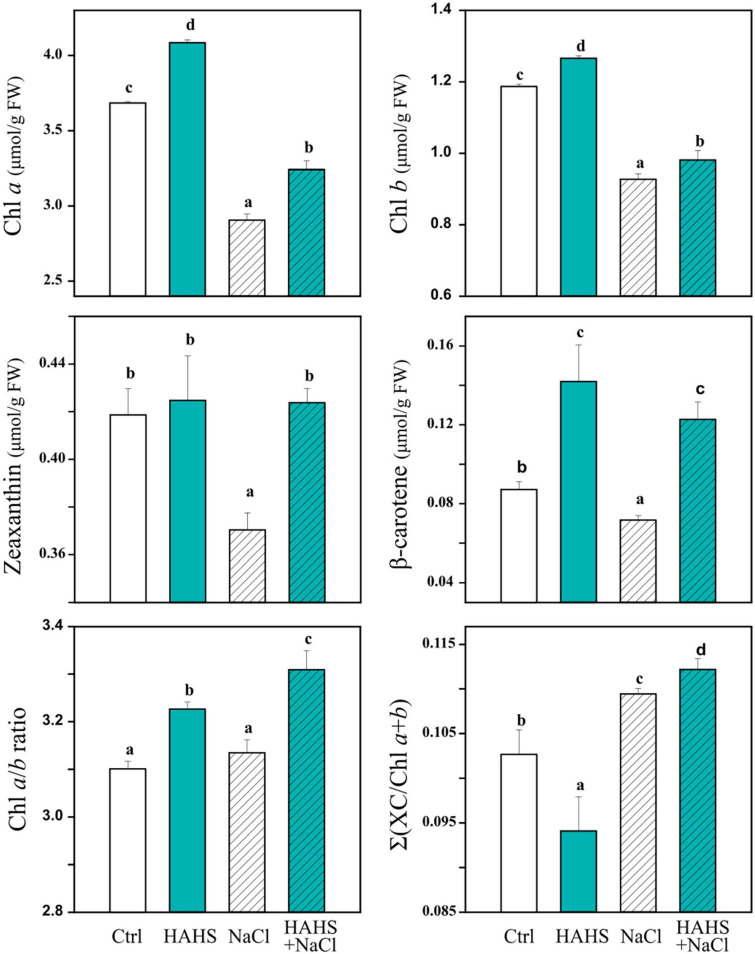
*Salinispora arenicola* induces changes in photosynthetically related pigments under normal and saline conditions. Plants (*n* = 4) 39 days after germination (DAG) were grown with either normal irrigation (white boxes) or saline irrigation (NaCl; gray boxes) and inoculated with a habitat-adapted heterologous symbiont (HAHS; B, turquoise) or not (control; C, black). Statistical significance was determined separately for each irrigation scheme using a univariate analysis of variance (ANOVA; *p =* 0.001). Lowercase letters denote statistical differences (pairwise test) among conditions.

The photosynthetic changes suggested the activity of photosynthetic and photoprotective-related pigments and metabolites. Pigment composition (Figure 6) and metabolite content (Table 2) were assessed in plant leaves collected 1 day after the last saline irrigation treatment. An increase in light collector molecules (Chl *a* by 11% and Chl *b* by 6 %) was observed in HAHS-treated plants under salt-free irrigation (Figure 6 and Supplementary Table 3), which provides evidence of extended photosynthetic performance induced by the actinobacterium. The increase was higher in Chl *a* than Chl *b*, which was reflected in the Chl *a*/*b* ratio. Moreover, the Xanthophyl cycle pool size $\left[\sum \left(\frac{X\,C}{C\,h\,l\,a+b}\right)\right]$ was reduced by 8% in HAHS-treated plants compared to that of the control plants, which was also likely due to the higher content of chlorophyll molecules. The photoprotective pigment zeaxanthin, which his related to the light-regulated energy dissipation mechanism, was not affected, although the antioxidant molecule beta-carotene increased ∼60% in HAHS-treated plants.

**TABLE 2:** Proline, phenolics, antioxidant activity, and reducing sugars in tomato plant leaves.

	Ctrl	HAHS	NaCl	HAHS + NaCl
Proline	37.39 ± 1.53^a^	35.68 ± 1.14^a^	133.55 ± 3.23^b^	93.08 ± 1.46^c^
Total phenolics	5.03 ± 0.22^a^	4.75 ± 0.28^a^	5.47 ± 0.332^b^	5.30 ± 0.16^b^
DPPH antioxidant activity	3.64 ± 0.48^a^	3.17 ± 0.45^a^	6.50 ± 0.303^c^	5.94 ± 0.34^b^
Reducing sugars	41.67 ± 1.34^c^	35.34 ± 0.92^b^	27.33 ± 0.70^a^	33.27 ± 2.29^b^

DPPH is a water soluble, stable radical 2,2-diphenyl-1-picrylhydrazyl; DPPH Antioxidant activity (μg Trolox/g FW), proline (μg/mg FW), reducing sugars (mg/g FW), total phenolics (mg GAE/g FW), and flavonoids (mg/g FW) in six-week-old tomato plant leaves. Ctrl = control, habitat-adapted heterologous symbiont (HAHS)-free; HAHS = S. arenicola; NaCl = HAHS-free and NaCl (100 mM); and HAHS+NaCl = S. arenicola and NaCl (100 mM). Values are mean ± standard deviation (n = 6). Different letters indicate significant differences according to Tukey tests (P < 0.05).

In HAHS-free plants, salt stress caused a significant reduction in all analyzed pigments [chl *a* (∼21%), chl *b* (∼22%), Zeaxanthin (∼11%), and Beta-carotene (∼19%)] and on the xanthophyll cycle pool size (∼16%). In contrast, treatment with HAHS prior to saline stress partially lessened the reduction in light collector molecules (chl *a* down to 12% and chl *b* to 17%) and the xanthophyll cycle pool (down to 6%) with respect to those of the control conditions. Furthermore, the content of Zeaxanthin and Beta-carotene was enriched up to 2 and 40%, respectively, which suggests induced zeaxanthin-related photoprotective and beta-carotene-related antioxidant activity by the actinobacterium *S. arenicola*.

Antioxidant metabolites, such as proline and phenol, and DPPH antioxidant activity tended to increase in plants under salinity as a strategy to mitigate stress by reducing the cellular osmotic potential and free radical scavenging. We found an increase in all of these parameters after saline irrigation. However, the increase was onefold lower in HAHS-treated plants than in untreated plants for both proline and DPPH (Table 2). This result implies that the symbiotic bacteria may reduce oxidative and osmotic stress and thus alleviate responses upstream, perhaps by inducing cation-transporters or reducing ROS formation.

Studies have also reported changes in sugar accumulation after saline stress (Yin et al., 2010), which are likely a consequence of a metabolic reconfiguration and redistribution of resources or an effect of photosynthetic changes. We observed a significant reduction in the content of reducing sugars (∼50%) when HAHS-free plants were treated with saline water (Table 2). This reduction was not observed in HAHS-treated plants. Sugar content reduction in untreated plants may be due to the observed decrease in photosynthesis (El-Beltagi et al., 2017). Overall, metabolic changes suggest that the symbiotic bacteria may lessen oxidative and osmotic stress and thereby protect plant metabolism.

### *Salinispora arenicola* modulates salinity responsive gene expression

**FIGURE 7:**
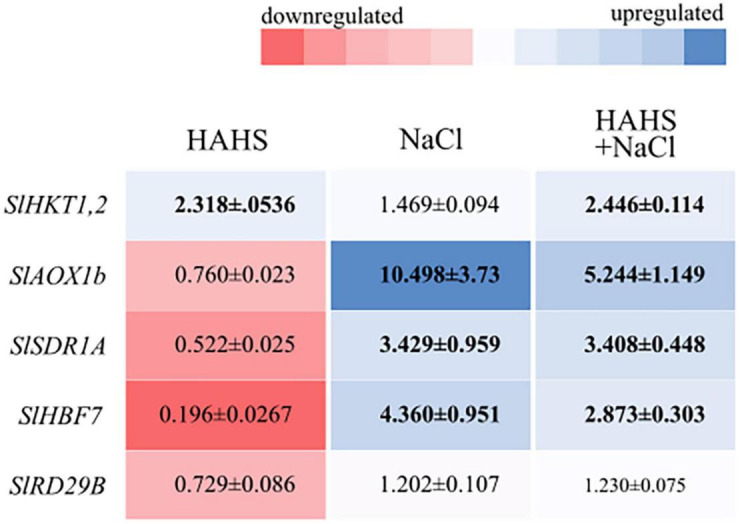
*Salinispora arenicola* modulates the expression of saline response genes in tomato plant leaves. Shade plot showing up- (blue) or downregulated (red) saline responsive genes in plants in the habitat-adapted heterologous symbiont (HAHS), HAHS + NaCl, and NaCl treatments. Gene expression was normalized to control expression. Black numbers inside the colored blocks indicate changes in expression at least 50% lower or higher than that of the control.

To obtain insights into the possible mechanism responsible for alleviating saline stress, the expression of the following five genes was assessed: *SlHBF7*, a transcription factor regulator of salt tolerance; *SlRD29B*, a gene involved in early responses to desiccation; *SlSDR1A*, a responsive gene involved in ABA biosynthesis; *SlHKT1,2*, a cation transmembrane transporter involved in Na^+^/K^+^ homeostasis; and *SlAOX1b*, a gene related with oxidative stress-related genes and ROS scavenging (Figure 7). Most of the genes were upregulated under salt stress conditions, with or without bacteria, except *SlRD29B*, which did not show any changes in expression. Interestingly, the expression of *SlHKT1,2* increased in HAHS-treated plants regardless of whether or not they were subjected to salt stress. *SlHKT1,2* encodes a Na^+^/K^+^ transporter and plays a crucial role in salinity tolerance mechanisms by alleviating osmotic stress. On the other hand, *SlAOX1b* and *SlHBF7* decreased their expression by half under salt stress conditions in HAHS-treated plants when compared to that of control plants under the same stress conditions. Therefore, HAHS treatment may induce changes that prime plants to better respond to salt-induced osmotic stress to avoid oxidative stress.

## Discussion

The indiscriminate use of agrochemicals coupled with the projected increases in population growth and soil salinity are expected to negatively impact the native microbiota of soils, and thus sustainable alternatives must be explored for plant agriculture. In this study, we show a growth-promoting and induced saline resilience effect in tomato plants by the marine actinobacteria *S. arenicola*. This actinobacterium established an endophytic symbiosis with tomato plant roots (Figure 2). The presence of this actinobacteria on tomato seeds relieved the inhibitory effect of salt on tomato germination and enhanced tomato plant growth under normal irrigation conditions (Figures 3, 4). Although salt-induced growth inhibition was still observed with HAHS-treated tomato plants, we observed enhanced quantum yield and photoprotective mechanisms (Figure 5). These changes appeared with increased quantities of photosynthetic pigments like Chl *a*, Chl *b*, β-carotene, and zeaxanthin along with increased amounts of reducing-sugars compared to those present in HAHS-free plants, which suggests improved photoprotection and a specific NPQ mechanism at play. Interestingly, we observed reductions in proline osmolyte and antioxidant activity in plant leaf tissues compared to that of the control plants under saline conditions (Figure 6 and Table 2), which implies that the bacterial treatment might ameliorate the underlying causes that induce these responses. Furthermore, after scouting for the possible mechanisms involved in these responses based on gene expression, only two genes with significant differences in expression between HAHS-treated and HAHS-free plants were identified (Figure 7). The expression of *SlHKT1,2*, which is involved in ion-transporter encoding, was found to be significantly higher in HAHS-treated plants than in HAHS-free plants under both normal and saline conditions. *SlHKT1,2* expression may explain the ameliorated responses in HAHS-treated plants to salt stress by restoring the K^+^/Na^+^ balance and relieving osmotic stress.

Microscopic analysis showed that *S. arenicola* established a stable symbiotic interaction with tomato plants (Figure 2). Interestingly, this interaction was observed to change with the salinity conditions of the substrate. Under salt-free conditions, *S. arenicola* was found on epithermal roots cells, while we observed them mainly distributed on root hairs under saline conditions. Both epidermal root cells and root hairs have been recognized as active interaction sites for microbes. The roles of root hairs in plant exudation in part determine the growth of symbiotic bacteria (Mercado-Blanco and Prieto, 2012). Furthermore, an evaluation of the effect of saline stress conditions on root exudation patterns in barley plants by Knipfer et al. (2021) revealed higher water exchange by exudation in the root hair region compared to the mature region (epidermal and endodermal cells) under saline stress, whereas higher water exchange occurs in mature regions in the absence of saline stress. Similar phenomena could occur in tomato roots under saline stress, with an increase in exudation in the root hair region that produces chemotaxis of *S. arenicola* and improves colonization in this region. Moreover, *Salinispora* is an obligate halophile (Mincer et al., 2002). Therefore, it is also possible that *S. arenicola* could migrate out of the plants or to more superficial cells, such as root hairs, in salty soil. In non-salty soil, the bacteria would likely prefer deeper cells to escape low-salinity soil. However, it would be interesting to further explore and define the specific reasons behind this behavior.

Tomato seeds exposed to salt stress showed delayed germination and a reduction in the percentage of germinated seeds. Salt stress disrupts germination by decreasing water uptake due to changes in the hydraulic potential or the activity of phytohormones, such as gibberellins (GAs) and abscisic acid (ABA; Liu et al., 2018). However, HAHS-treated seeds under saline conditions showed the same overall germination percentage as that of control plants, although with a slight delay. GAs activate amylase enzymes that make endosperm energy available in the embryo for germination; however, salinity is known to affect amylase activity (Liu et al., 2018). The recovery of the germination percentage in HAHS-treated seeds may be explained by the high production of saline-stable glycosyl hydrolases by *S. areniola*, including α-amylase, endoglucanases, and endomanases, which are seen in the *S. arenicola* genome (CNS-205, T00613; Penn and Jensen, 2012) and published in the Kyoto Encyclopedia of Genes and Genomes database^2^. Therefore, even if GAs are not produced in sufficient concentrations due to low water uptake during imbibition under saline conditions, the enzymes necessary for germination could be provided by the HAHS *S. arenicola*.

**FIGURE 8:**
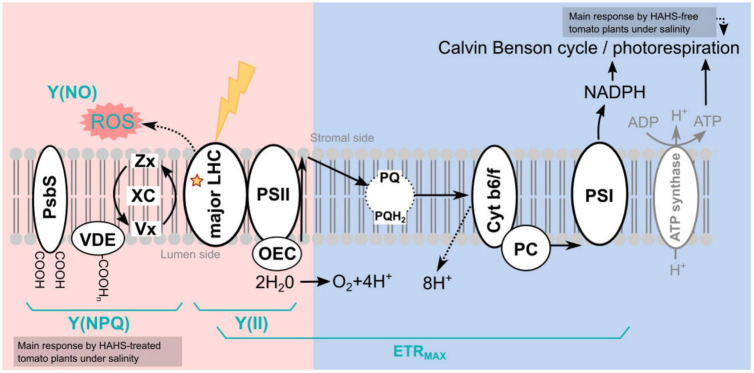
Model of habitat-adapted heterologous symbiont (HAHS)-induced changes in the light reactions of photosynthesis and associated alternative electron transfer pathways. The light energy distribution (pink side) and electron transport (blue side) of the light reactions of photosynthesis are shown. In the control plants, the photochemical energy flows to NADPH, mainly *via* the linear electron flow (LEF) pathway. Reduced Y(NO) and increased Y(II) indicate a lower probability of non-regulated dissipation but a higher probability of energy conduction to photochemistry as observed for HAHS-treated plants. As a result, plants exhibited higher F_V_/F_M_ values and high plant growth. An increase in Chl content, the Chl *a*/*b* ratio, and beta-carotene but a reduction of XC/Chl *a*+*b* suggests a reduction of antenna size and an increase of PSII reaction centers in agreement with the increase in photosynthetic performance. High ETR_MAX_ with a small antenna and no PSII damage indicates highly efficient energy consumption in the Calvin cycle induced by HAHS interaction. Plants under salt stress without HAHS did not exhibit differences in Y(NO) or Y(NPQ) dissipation, indicating that no induced protective mechanism was present in the antenna. However, a significant increase in Y(II) linked to lower plant size was found, suggesting that a salt-induced mechanism in tomato plants without HAHS is related to the activation of alternative sinks such as photorespiration. Induced stomatal closure due to saline stress reduces CO_2_/O_2_ ratio increases the photorespiration probability. Photorespiration reduces the energy destined for photosynthetic Calvin Benson cycle, thereby affecting plant growth. In plants treated with HAHS under saline conditions, the HAHS protects plant photosynthesis by increasing the regulated thermal energy dissipation Y(NPQ) pathway, which decreases the potential for ROS formation in the LHCII antenna. This is also reflected in high F_V_/F_M_ values compared to those of the control and control + NaCl plants. PsBS, pigment-binding protein associated with photosystem II (PSII); VDE, Violaxanthin De-Epoxidase; XC, Xanthophyll cycle pigments (Zx+Ax+Vx); Zx, Zeaxanthin; Ax; Antheraxanthin, Vx, Violaxanthin; LHC, Light-Harvesting Complex; PSII, Photosystem II; OEC, oxygen-evolving complex; PQ, Plastoquinone; PQH, Plastoquinol; AOX, Alternative Oxidase; Cyt b6/f, Cytochrome b6/f; PC, Plastocyanin; PSI, Photosystem I; ROS, Reactive oxygen species; ETR_MAX_, Maximum electron transfer rate; Y(NO), Quantum yield of non-regulated heat dissipation and fluorescence emission; Y(II), Effective photochemical quantum yield of PSII; Y(NPQ), Quantum yield of light-induced non-photochemical fluorescence quenching.

Salt exposure also affected the growth of tomato plants, independent of the bacterial treatment. However, HAHS-treated plants were healthier, with thicker stems, greener leaves, and fewer senescent leaves than those of HAHS-free plants. It is possible that the initial effect on growth and perhaps nutrition by HAHS treatment prior to saline irrigation may have prepared the plant to better cope with salt stress conditions. This is consistent with the onset of arrested growth. However, we observed increased photoprotection parameters, such as NPQ, and a reduction in Y(NO) and ETR_MAX_ in HAHS-treated plants under salt stress conditions. ETR_MAX_ increased in HAHS-free plants under salt stress conditions, although this does not seem to be consistent with the observed arrested growth. It could be argued that the electron flow in these plants was directed toward an alternative electron sink pathway, such as photorespiration (Figure 8). Photorespiration is considered to be a significant electron sink pathway for C3 plants under saline stress (Di Martino et al., 1999; Hossain and Dietz, 2016). Absicic acid-mediated stomata closure is one of the first responses to salt stress (Zhao et al., 2021). Stomata closure decreases the availability of CO_2_ in leaves and increases the probability of RuBP oxygenation by Rubisco, which leads to photorespiration (Kalaji et al., 2011). Photorespiration is an energy-expensive process that affects plant growth (Walker et al., 2016). Therefore, although an increase in ETR_MAX_ was observed in NaCl-treated plants, it does not necessarily translate to increased photosynthesis and growth. HAHS-treated plants seem to prevent this scenario by redirecting quantum energy to dissipation mechanisms, as evidenced by the increase in NPQ, which is consistent with the increase in zeaxanthin pigment. The onset of this NPQ mechanism (Figure 8) allows the plant to reduce PSII pressure and prevent oxidative damage through the reduction of the un-regulated dissipation of energy [Y(NO)], which may result in ROS production at the photosynthetic antenna.

To avoid damage caused by the overproduction of ROS, plants employ an arsenal of antioxidant defense mechanisms, one of which involves the synthesis of molecules with antioxidant properties, such as polyphenols (Salem et al., 2014; Zhao et al., 2015). Antioxidant production is often observed as a plant tolerance mechanism to cope with salt stress (Soares et al., 2019; Akyol et al., 2020). Salt stress affects the electron transport chain, which may favor the formation of potentially harmful ROS that are then scavenged by antioxidants (Ahanger et al., 2017; Numan et al., 2018). Inoculation with several plant growth-promoting rhizobacteria (PGPR), such as *Sphingobacterium* BHU-AV3 in tomato plants, increases phenols and antioxidative enzymes and directly alleviates the induced oxidative stress (Kohler et al., 2009; Hashem et al., 2016; Vaishnav et al., 2020). As expected, phenols and the overall antioxidant activity in plant leaf tissues increased with salt stress in tomato plants. However, no significant changes were observed in phenols or antioxidant activity between HAHS-treated and HAHS-free plants (Table 2) that would improve the performance of the HAHS-treated plants in our experiment. It is likely that the higher NPQ levels observed in HAHS-treated plants under salt stress may contribute to avoiding ROS formation and thus reducing oxidative stress beforehand. Therefore, although antioxidant activity and metabolites were equivalent in plants with or without HAHS treatment, oxidative stress may have been reduced in HAHS-treated plants. Further experiments that analyze ROS production, ROS half-life times, and other antioxidant metabolites are needed to answer this question.

The presence of bacteria alone induced a drop in reducing sugars even though ETR_MAX_ was enhanced. An increase in ETR_MAX_ suggests an increase in sugar production. However, it is likely that the rate of sugar consumption also increased in HAHS-treated plants, with sugars being redirected to growth and development (as suggested by the high shoot and root lengths and the number of branches) to sustain the symbiotic relationship with the bacteria. The symbiotic consumption of photosynthetically produced sugars has been demonstrated for other root symbiotic interactions, such as mycorrhizal interactions (Schweiger et al., 2014). On the other hand, salt-stress conditions lowered the sugar levels in HAHS-free plants. This was also observed in ETR_MAX_ levels, which were increased by salt stress in HAHS-free plants. However, considering that growth was inhibited, the flow of the electrons at ETR_MAX_ may follow alternative electron sink pathways. Moreover, the rapid secretion of photosynthetically produced molecules other than sugars, such as osmolytes, can lead to high ETR_MAX_ but low growth rates (Demmig-Adams et al., 2017). Interestingly, salt stress conditions had no significant effect on reducing sugars in HAHS-treated plants. This may be due to a reduction in ETR_MAX_ by redirecting quantum energy to dissipative NPQ mechanisms and a decrease in the sugar used, as suggested by the reduced growth. This coordinated response may favor the plant by avoiding energy waste and oxidative stress.

Proline accumulation in the leaves and roots is considered to be a salt-sensitive trait in tomato plants that may be used to select varieties with different degrees of tolerance (Gharsallah et al., 2016). Many reports have shown that the proline concentration increases in the shoots of plants grown under saline conditions (Vicente et al., 2004; Amini and Ehsanpour, 2005; Roy et al., 2014). Plants accumulate proline as a non-toxic and protective compatible solute, which participates in cytosolic osmotic adjustments and balances osmotic pressure differences between the cytosol and vacuoles (Parida and Das, 2005; Munns and Tester, 2008; Kamanga et al., 2020). Additionally, proline acts as an energy supplier and a signaling/regulatory molecule that can activate responses involved in the adaptation process, which helps to improve salinity tolerance (Maggio et al., 2002). In agreement with this, our experiments showed increased proline levels when plants were exposed to salt stress conditions, although this increase was lower in HAHS-treated plants (Table 2). As with any stimuli response mechanism, a quantitative level of damage or impact must induce a protective response of a similar scale. In this case, a lower response in HAHS-treated tomatoes does not necessarily mean a lower capacity to protect against the damaging effects of salt, as suggested by the observed growth and photosynthetic performance (Figures 4, 5). Instead, this could be due to lower adverse effects produced by Na^+^ ions. A potential hypothesis is that the activation of ion transporters ameliorates some of the adverse effects of Na^+^. Which is line with many other studies that have shown that the activation of HKT1 transporters under saline stress conditions increases salinity tolerance in plants (Rodríguez-Navarro and Rubio, 2006). This aligns well with the observed overexpression of *SlHKT1,2* in HAHS-treated plants with regular or saline irrigation (Figure 7). The increased expression of *SlHKT1,2* in HAHS-treated plants, even in the absence of salt stress, could serve as a mechanism that prepares the plants to withstand stress by ameliorating other stress responses. In agreement with this, HKT1 induction has been proposed as a protective mechanism to salinity stress conditions in halophytes (Rodríguez-Navarro and Rubio, 2006) or in salt sensitive plants, where growth promoting bacteria (Zhang et al., 2008) and synthetic bacterial community (Schmitz et al., 2022) induced HKT1 expression. However, *SlHKT1,2* gene behavior in response to HAHS even in salty or regular conditions, needs further investigation. Additionally, the decreased expression of *SlAOX1b* and *SlHBF7* under saline conditions in HAHS-treated plants compared with those of the control plants indicates lower damage caused by free radicals and other salt-related effects. *Salinispora* is endemic to marine ecosystems, and *Salinispora* strains offer a vast array of potential benefits to their ubiquitous hosts. These strains are recognized for their antimicrobial activity (Cardoso-Martínez et al., 2015; Chase et al., 2021), ability to modify microbiotas (Tuttle et al., 2019), and ability to degrade recalcitrant materials. The particular strain used in this study was isolated from corals. However, many other strains have been obtained from other marine sources, including ascidians (Steinert et al., 2015), seaweeds (Jensen et al., 2005), soft corals (Louka et al., 2022), and sponges (Kim et al., 2005), which suggests that *Salinispora* is a ubiquitous symbiont. However, we were surprised to find that this actinobacterium was able to sustain a stable interaction with a terrestrial plant, although *S. arenicola* is closely related to several terrestrial actinobacteria (Trujillo et al., 2014). Thus, the ability to sustain this interaction may be due to a conserved mechanism present among terrestrial or marine actinobacteria or an evolutionary memory in *S. arenicola* metabolism that allows it to interact with terrestrial plants. A better understanding of the interactions between *S. arenicola* and terrestrial plants may allow us to identify the fundamental mechanisms necessary for sustaining symbiotic relationships, promoting growth, and alleviating saline stress. We define this interaction as a habitat-adapted heterologous symbiosis following the guidelines of Rodriguez et al. (2008). However, ecological interactions are not always positive. For example, Chhun et al. (2021) demonstrated that *S. arenicola* could inhibit the growth of some marine phototrophs. Therefore, it is essential to further study actinobacteria interactions to mitigate any potential side effects in terrestrial plants.

## Data availability statement

The original contributions presented in this study are included in the article/Supplementary material, further inquiries can be directed to the corresponding author.

## Author contributions

HO-A, AB-E, and RH-H designed and formulated the concept of the study. AB-E, RH-H, RP-R, CS-H, PP-S, EJ-C, OP, FC, ML-G, MH-O, LM-M, and HO-A performed part of the measurements in laboratory and interpreted part of the results. AB-E, IM-C, FR-Z, and HO-A carried out the analysis of the data. AB-E, RH-H, IM-C, and HO-A draft the manuscript with contributions of CS-H and PP-S. All authors approved the final manuscript.

## Footnotes

1
http://www.ncb.nlm.nih.gov/genbank/index.html

2
https://www.genome.jp/kegg/kegg2.html
