# Supplementary material for: Habitat-adapted heterologous symbiont Salinispora arenicola promotes growth and alleviates salt stress in tomato crop plants
Source: Front Plant Sci. 2022 Aug 8;13:920881. doi: 10.3389/fpls.2022.920881 (PMC9393590; doi:10.3389/fpls.2022.920881)
Supplement: Supplementary file 1 [file Data_Sheet_1.docx]

Supplementary Material

**Supplementary Table S1. Photosynthetic parameters**

| **Abbreviation** | **Parameter description** | **Formula** | **Reference** |
| --- | --- | --- | --- |
| *F_v_/F_M_* | Maximum photochemical quantum yield of PSII | $\frac{F_{V}}{F_{M}}= \frac{F_{M}- F_{O}}{F_{M}}$ | Kitajima and Butler, 1975 |
| ETR | Electron transfer rate | $ETR=\frac{F_{M}^{´}-F}{F_{M}} (PAR) (0.5)(0.84)$ | White and Critchley, 1999 |
| NPQ | Stern-Volmer type non-photochemical fluorescence quenching | $NPQ= \frac{F_{M}}{F_{M}^{´}}-1$ | Bilger and Björkman., 1990 |
| Y(II) | Effective photochemical quantum yield of PSII | $Y\left( II \right)=\frac{F_{M}^{´}-F}{F_{M}^{´}}$ | Genty et al., 1989 |
| Y(NO) | Quantum yield of non-regulated heat dissipation and fluorescence emission. *This quenching type does not require the presence of a trans- thylakoid ΔpH and zeaxanthin* | $Y \left( NO \right)= \frac{F}{F_{M}}$ | Genty et al., 1996 |
| Y(NPQ) | Quantum yield of light-induced non-photochemical fluorescence quenching | $Y\left( NPQ \right)= \frac{F}{F_{M}^{´}}- \frac{F}{F_{M}}$ | Genty et al., 1996 |

**Supplementary Table S2. Oligo sequences used in qPCR**

| **Gene ID** | **Reverse primer** | **Forward primer** | **Function** |
| --- | --- | --- | --- |
| *SlAOX1b /* Solyc08g075550 | ATGGCCTGAAGCTGTTCCACTTCC | CACACACTGATGCAACCAACGC | Alternative oxidase Defense and ROS scavenging |
| *SlSDR1A /* Solyc12g056600 | TATACTACCGTTGCCGCACTTG | TGCTGCTAGAGTGATGATTTCGAG | Short chain alcohol dehydrogenase/reductase ABA biosynthesis |
| *SlHKT1,2 /* Solyc07g014680 | TGTCTTTCTCCACAACACAAATAAG | GGGAAGAAGCTTAATGGAGTACA | HKT sodium transporter |
| *SlHB7 /* Solyc01g096320 | CAGTAGCAGTTGCTTCAGCAGTC | CTGAACCTCCTCGCGTTGTTAC | Homeobox 7 stress and salinity induced TF |
| *SlRD29B* / Solyc03g025810 | AACAATGAGGAGGCAGGAGTTAGC | GCTTCCGGAACATCGTTCACAC | Responsive to desication 29B stress and salinity responsive gene |
| *SlGAPDH* / Solyc04g009030 | ACTGGTGCTGCTAAGGCTGT | ACAAGGTCCACAACCGAGAC | Reference gene |
| *SlEF1α* / Solyc06g005060.2.1 | TGGAAACGGATATGCCCCTG | TGGGCTTGGTGGGAATCATC | Reference gene |

Primers for *SlGADPH* and salinity responsive genes were reported by Devkar et al., 2020 and *SlEF1α* by Wang et al., 2020.

**Supplementary Table S3. Photosynthetic plant pigments read in tomato plants under different treatments**

|  | Ctrl | HASH | NaCl | HASH+NaCl |
| --- | --- | --- | --- | --- |
| Chl *a* (µmol g) | 3.689 ± 0.005**^c^** | 4.085 ± 0.020**^d^** | 2.906 ± 0.039**^a^** | 3.246 ± 0.053**^b^** |
| Chl *b* (µmol g) | 1.189 ± 0.005**^c^** | 1.266 ± 0.007**^d^** | 0.927 ± 0.015**^a^** | 0.981 ± 0.026**^b^** |
| Vx (µmol g) | 0.062 ± 0.002**^b^** | 0.060 ± 0.003**^b^** | 0.037 ± 0.002**^a^** | 0.038 ± 0.001**^a^** |
| Ax (µmol g) | 0.020 ± 0.003**^b^** | 0.019 ± 0.001**^b^** | 0.012 ± 0.000**^a^** | 0.011 ± 0.000**^a^** |
| Zx (µmol g) | 0.419 ± 0.011**^b^** | 0.425 ± 0.019**^b^** | 0.370 ± 0.007**^a^** | 0.423 ± 0.006**^b^** |
| ƩXC (Vx+Ax+Zx) (µmol g) | 0.501 ± 0.013**^c^** | 0.503 ± 0.020**^c^** | 0.419 ± 0.005**^a^** | 0.474 ± 0.006**^b^** |

Chlorophyll *a* (Chl *a*), Chlorophyll *b* (Chl *b*), Antheraxanthin (Ax), Violaxanthin (Vx), Zeaxanthin (Zx), ƩXC, xanthophyll cycle pigment pool from the sum of antheraxanthin, violaxanthin and zeaxanthin (µmol/g); Ctrl, plants under control conditions; HAHS, plants growth from bacteria embedded roots inoculated on day 15; NaCl, plants watered from the day 30 to 38 with increasing NaCl concentrations; HAHS+NaCl, Plants growth from bacteria embedded roots and watered from the day 30 to 38 with increasing NaCl concentrations. Values are mean ± standard deviation (n=4). In μmol g

**Reference**

Bilger, W., and Björkman, O. (1990). Role of xanthophyll cycle in photoprotection elucidated by measurements of light-induced absorbance changes, fluorescence and photosynthesis in leaves of *Hedera canariensis*. *Photosynth. Res.* 25, 173-185. doi.org/10.1007/BF00033159.

Devkar, V., Thirumalaikumar, V. P., Xue, G. P., Vallarino J. G., Tureckova, V., Strnad, M., et al. (2020). Multifaceted regulatory function of tomato SlTAF1 in the response to salinity stress. *New Phytol.* 225, 1681-1698. doi: 10.1111/nph.16247.

Genty, B., Briantais, J. M., and Baker, N. R. (1989). The relationship between the quantum yield of photosynthetic electron transport and quenching of chlorophyll fluorescence. *Biochim. Biophys. Acta*. 990, 87-92. doi: 10.1016/S0304-4165(89)80016-9.

Genty, B., Harbinson, J. M., Cailly, A. L., and Rizza, F. (1996). Fate of excitation at PS II in leaves: the non-photochemical side. Presented at The Third BBSRC Robert Hill Symposium on Photosynthesis, March 31 to April 3, 1996, University of Sheffield, Department of Molecular Biology and Biotechnology, Western Bank, Sheffield, UK, abstract no. P28.

Kitajima, M., and Butler, W. L. (1975). Quenching of chlorophyll fluorescence and primary photochemistry in chloroplasts by dibromothymoquinone. *Biochim. Biophys.* *Acta*. 376, 105-115. doi: 10.1016/0005-2728(75)90209-1.

White, A. J., Critchley, C. (1999). Rapid light curves: A new fluorescence method to assess the state of the photosynthetic apparatus. *Photosyn. Res.* 59, 63-72. doi: 10.1023/A:1006188004189
